# Endoscopic Step‐up Approach for Walled‐off Necrosis After Acute Pancreatitis

**DOI:** 10.1002/deo2.70188

**Published:** 2025-08-25

**Authors:** Shuntaro Mukai, Atsushi Sofuni, Takayoshi Tsuchiya, Reina Tanaka, Ryosuke Tonozuka, Yukitoshi Matsunami, Kazumasa Nagai, Hiroyuki Kojima, Hirohito Minami, Noriyuki Hirakawa, Kyoko Asano, Kento Shionoya, Kazuki Hama, Takao Itoi

**Affiliations:** ^1^ Department of Gastroenterology and Hepatology Tokyo Medical University Tokyo Japan

**Keywords:** endoscopic necrosectomy, endoscopic ultrasound‐guided pancreatic fluid collection drainage, lumen‐apposing metal stent, step‐up approach, walled‐off necrosis

## Abstract

This review outlines current interventional strategies for treating symptomatic walled‐off necrosis (WON) after necrotizing pancreatitis. Mortality from acute pancreatitis has improved, but late mortality, particularly from infected necrosis, remains a challenge. WON requires invasive treatment in cases of infection or symptoms. A step‐up approach is recommended, in which minimally invasive drainage is performed first, followed by more invasive treatments if the effect is insufficient. Among these, an endoscopic step‐up approach mainly consisting of transmural treatment using endoscopic ultrasound‐guided drainage and endoscopic necrosectomy (EN) has been reported with favorable outcomes. The use of lumen‐apposing metal stents (LAMSs) has enhanced drainage efficiency and facilitated EN, although bleeding and stent‐related adverse events remain concerns. Recent techniques such as multiple transluminal gateways and transcystic drainage have improved outcomes for complex, multilocular WON. With the introduction of the LAMS and additional endoscopic drainage techniques, most cases can be successfully treated with endoscopic therapy alone. However, endoscopic treatment alone has limitations for lesions spreading to the pelvic cavity, and a combination of percutaneous treatment or surgical treatment should be considered.

## 1 Introduction

The results of the 2016 nationwide epidemiological survey of acute pancreatitis in Japan showed that the mortality rate for acute pancreatitis overall had improved from 2.6% to 1.8%, and the mortality rate for severe pancreatitis had improved from 10.1% to 6.1%, compared to the 2011 survey. However, the late mortality rate after 2 weeks of onset has not changed from 3.5% to 3.4% [1]. One of the reasons for this is that there has been no improvement in the mortality rate of infected pancreatic necrosis (a general term for acute necrotic collection [ANC] and walled‐off necrosis [WON]), which is a late local complication associated with necrotizing pancreatitis. The mortality rate for cases of acute pancreatitis that didn't develop WON was 1.3%, while the mortality rate for cases that developed WON was 6.7%. Therefore, one of the issues for further improving the treatment outcomes for acute pancreatitis is the treatment of infected pancreatic necrosis.

Infected WON is often difficult to control with conservative treatment using antibiotics alone, and can lead to bacteremia and multiple organ failure, so invasive treatment is often required. The open surgical necrosectomy that was previously performed was highly invasive, and the high morbidity and mortality rates were an issue [2]. Therefore, recently, minimally invasive endoscopic treatment using a step‐up approach that focuses on endoscopic treatment via the intestinal tract, using endoscopic ultrasound‐guided pancreatic fluid collection drainage (EUS‐PFD) followed by endoscopic necrosectomy (EN), has become the main approach, and it is also recommended in the guidelines on the management of acute pancreatitis [3]. However, the size, morphology, amount of necrotic tissue contained, and degree of pancreatic duct disruption differ greatly between cases of WON, and the optimal treatment strategy also differs. In this review, we summarize the tailored treatment strategies for infected WON depending on the case.

### 1.1 Step‐up Approach

The conventional open surgical necrosectomy is a highly invasive treatment with a high risk of morbidity in patients with poor general condition. Reports showed a 55% morbidity rate and a 14% mortality rate, so a less invasive and more effective treatment was required [2]. Therefore, a step‐up approach was developed, starting with less invasive drainage and adding more invasive treatment as necessary. In 2010, a randomized controlled trial (RCT) was reported from the Netherlands comparing a step‐up approach, in which percutaneous or endoscopic drainage was performed first, and less invasive necrosectomy via retroperitoneal approach was added as needed, with open necrosectomy [4]. The results showed that the step‐up approach was less invasive and more useful. Following this, further research led to a consensus that the less invasive step‐up approach should be the first choice in the treatment of infected WON [5, 6, 7, 8].

This step‐up approach can be broadly divided into two types. One is an endoscopic step‐up approach that mainly uses endoscopic treatment, including EUS‐PFD and subsequent EN. The other is a surgical step‐up approach that uses percutaneous drainage and subsequent video‐assisted retroperitoneal debridement (VARD) with laparoscopic assistance. An RCT comparing the two step‐up approaches reported in 2012 from Germany showed that the endoscopic approach had significantly fewer adverse events and postoperative complications, and although not significant, the overall mortality was also lower (10% vs. 40%) [9]. In recent RCTs, although there was no significant difference in mortality, the endoscopic group had fewer serious adverse events, particularly fewer cases of refractory skin fistula, and shorter hospital stays [10, 11]. A meta‐analysis of three RCTs was also presented in the Japanese guidelines, showing that the hospital stay was significantly shorter in the endoscopic group, and the incidence of major adverse events and mortality tended to be lower, though not significantly [3].

As a result of these studies, the endoscopic step‐up approach has become the mainstream strategy for treating infected WON. However, the surgical step‐up approach remains useful in cases where endoscopic access is difficult, such as WON cavities extending to the pelvic area or right colonic sulcus. It is essential to select the optimal approach for each case, rather than being limited to a single method.

## 2 Timing of Intervention Treatment Introduction

Regarding the timing of invasive intervention therapy, it has been recommended that it be performed during the phase of encapsulated WON (4 weeks or more after onset), as reports have shown significantly higher mortality and morbidity rates when open necrosectomy is conducted during the phase of insufficiently encapsulated ANC (less than 4 weeks after onset) [12]. In a prospective study by van Santvoort et al., the longer the time to surgical intervention, the lower the risk of adverse events (0–14 days: 72%, 15–29 days: 57%, 30 days or more: 39%) [13]. However, as mentioned earlier, recent trends favor a step‐up approach starting with minimally invasive drainage, and the situation differs due to advancements in techniques and equipment. During conservative treatment with antibiotics, some patients deteriorate due to complications such as acute respiratory distress syndrome, disseminated intravascular coagulation, or intra‐cavity hemorrhage, making further treatment difficult. Perforation of the surrounding gastrointestinal tract has also been observed due to inflammation and increased cavity pressure in WON [14, 15]. Notably, when WON perforates the colon, infection control becomes challenging even if drainage is successful [16]. Recent studies have compared treatment outcomes between early (within 4 weeks) and delayed (after 4 weeks) minimally invasive interventions. Although mortality tends to be higher in early intervention groups due to more severe cases, there is no significant difference in procedure‐related adverse event rates, supporting the feasibility of early intervention [17, 18, 19, 20]. If liquefaction and encapsulation are progressing on imaging, there is no need to wait 4 weeks to initiate drainage—it is crucial not to miss the appropriate timing for treatment.

### 2.1 EUS‐guided Pancreatic Fluid Collection Drainage

In 1975, drainage by cyst puncture under direct endoscopic visualization was first reported [21]. Later, EUS technology was developed, and in 1992, Grimm et al. first reported EUS‐PFD [22]. Since EUS allows selection of the shortest puncture route and avoidance of intervening blood vessels, puncture can be performed more safely. In fact, two RCTs reported that EUS‐PFD has a higher procedural success rate and a lower adverse event rate than direct endoscopic drainage [23, 24]. There have also been fatal adverse events due to serious bleeding in the direct endoscopic drainage group. With the development of techniques and devices, EUS‐PFD has become widespread worldwide, and is now considered a very useful and safe treatment, with a technical success rate of approximately 95% for lesions with little necrotic tissue, a clinical response rate of about 90%, and an adverse event rate of 0%–9% [25, 26]. Regarding the technique, the lesion is punctured under EUS, and a long guide wire is inserted. The tract is then dilated using a cautery or mechanical dilator and a 4–6 mm dilation balloon. The standard procedure is to place one or more 7Fr double‐pigtail plastic stents and a transnasal drainage tube for internal‐external drainage [27]. The effectiveness of EUS‐PFD for WON has also been reported in many studies. However, since WON often contains necrotic tissue, drainage is often insufficient, and the clinical response rate is reported to be around 40%–50% [28].

### 2.2 Endoscopic Necrosectomy

For WON containing a large amount of necrotic tissue, drainage is often not effective with EUS‐PFD alone, making infection control difficult. EN was then developed, involving insertion of an endoscope through a tract created by EUS‐PFD to remove infected necrotic tissue. It was first reported by Seifert et al. in 2000 [28]. Gardner et al. reported that the clinical success rate with EUS‐PFD alone for WON was 45%, but that adding EN improved the rate to 88% [29]. Since then, many facilities have reported this procedure, with a clinical success rate of 75%–91%, an adverse event rate of 26%–33%, and a mortality rate of 5.8%–11% in multicenter, large‐case studies in Germany, the U.S., and Japan [30, 31, 32]. These studies suggest that EN is more effective than EUS‐PFD alone and safer than open necrosectomy. In Japan, the JENIPaN study [30] reported a clinical success rate of 75% (median treatment period 21 days) for 57 cases of infected WON at 16 facilities, with an adverse event rate of 33% and a mortality rate of 11%.

**FIGURE 1:**
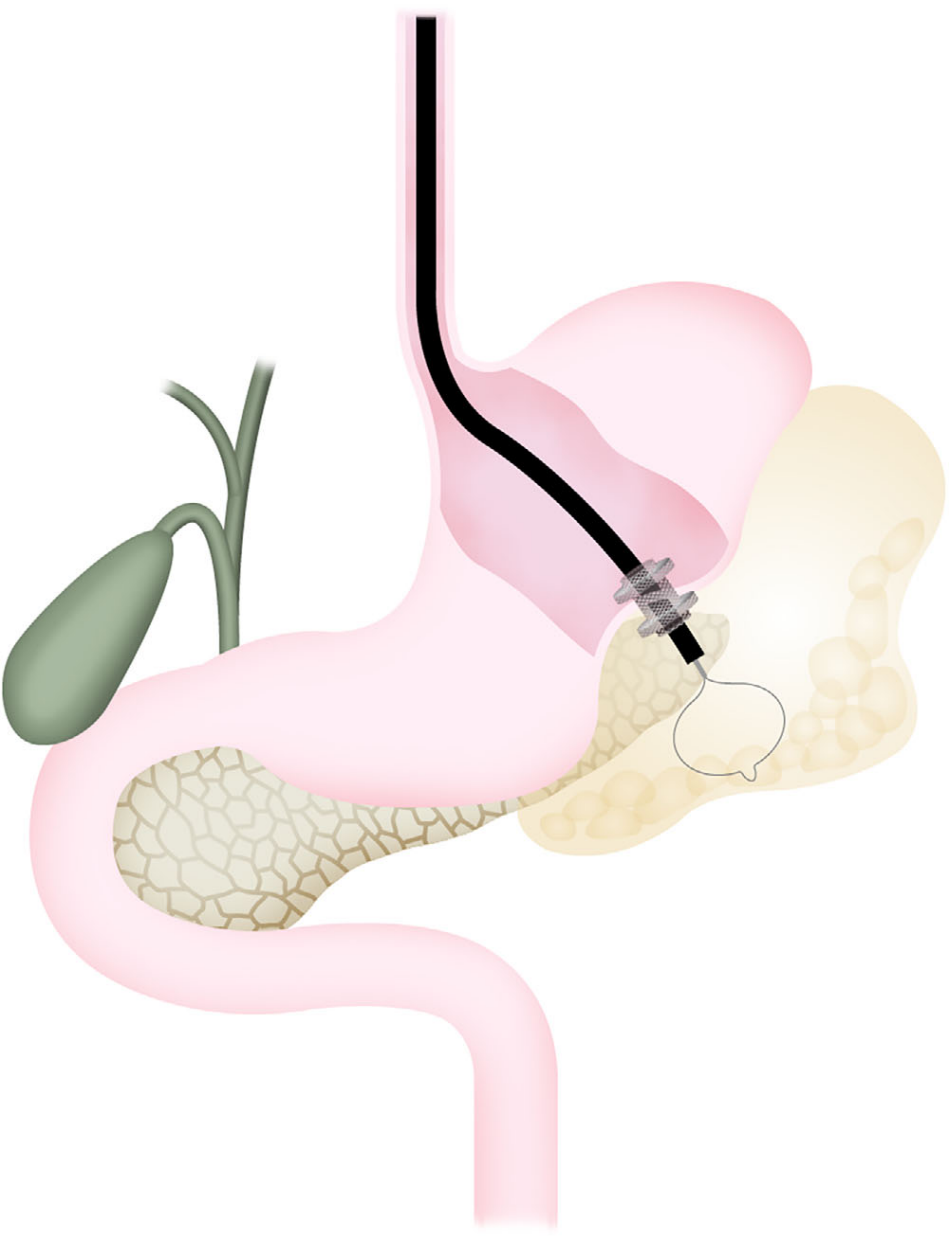
Schema showing additional endoscopic necrosectomy via a lumen‐apposing metal stent.

Technically, an endoscope is inserted, and a guide wire is placed into the WON through the tract. The fistula is then dilated using an 18–20 mm balloon, and the endoscope is gently advanced into the WON. The cavity is lavaged with saline, and necrotic tissue is gradually removed using a snare or forceps. When a large‐bore metal stent (described later) is used for EUS‐PFD, the endoscope can be inserted through the stent lumen without balloon dilation. This facilitates easy insertion and removal, improving necrosectomy efficiency (Figure 1). There is no established procedure for EN. As our procedure, we mainly use a 10 mm mini snare to remove necrotic tissue near the placed stent from the WON cavity into the stomach little by little, paying attention to damage to hidden blood vessels. At the end of the procedure, a naso‐drainage tube is placed in the deeper cavity, and a saline irrigation is performed during the procedure. This will make it easier to remove necrotic tissue during the next procedure, as the necrotic tissue in deeper areas will come to the front. Various techniques improving the efficacy of EN have been reported, such as low‐concentration hydrogen peroxide (0.1–0.3%) to separate necrotic tissue [33], a tunneling method to enhance efficiency [34], and balloon‐assisted enteroscopes to improve access to the pelvic cavity [35]. However, further development of dedicated devices and techniques is needed. Necrotic tissue removal should be done carefully to avoid vessel injury or perforation. Once granulation tissue appears, that area is considered sufficient. From a safety perspective, it is considered appropriate to perform the EN procedure twice a week for approximately one hour, but a longer procedure time may shorten the hospital stay. Evidence on this point is lacking, and the difficulty and risk of EN vary between cases, so treatment schedules must be tailored to each case. Given its invasiveness and risks, a step‐up approach—starting with drainage and determining the need for EN based on clinical course—has generally been favored. However, a recent RCT comparing upfront necrosectomy with a step‐up approach reported that upfront necrosectomy can be safe and may reduce the number of procedures and hospital stay. For WON with extensive necrosis, one‐stage necrosectomy (upfront EN) may be acceptable [36]. Conversely, some cases can be treated without EN by continuous saline irrigation (1 L/day) through the transnasal drainage tube placed with EUS‐PFD, so further research is needed regarding indications and methods of EN [37].

The most common adverse event associated with EN is bleeding. The results of the JENIPaN study in Japan also showed that most adverse events were related to bleeding. In particular, because aneurysm rupture can be fatal, contrast‐enhanced computed tomography (CT) should be evaluated before drainage and regularly during treatment. If an aneurysm is detected, it is better to first treat it using coil embolization by interventional radiology (IVR) if the patient's general condition is stable and drainage can be delayed [38]. For bleeding during the EN procedure, carefully check the bleeding point and try endoscopic hemostasis. For bleeding during tract balloon dilation, compression using a balloon or metal stent, and for vascular injury during necrosectomy, clipping, argon plasma coagulation, and coagulation with hemostatic forceps are useful. However, if bleeding occurs inside the WON cavity during the interval period, it is almost impossible to identify the bleeding point due to accumulating blood clots, and IVR is required. Therefore, EN should be performed at institutions with a well‐established IVR backup system. Air embolism can also be fatal, so CO_2_ must be used for insufflation. Excessive CO_2_ insufflation may cause WON rupture, and although rare, CO_2_ embolism can occur, so it should be avoided [39]. To prevent rupture, necrotic tissue should be removed gradually rather than proceeding deep in one session. Pneumoperitoneum or suspicious fluoroscopic signs of rupture should be checked during EN.

### 2.3 Endoscopic Step‐up Approach Using Large‐bore Metal Stents

Conventionally, it was common to place one or more double pigtail plastic stents(DPSs)using EUS‐PFD. Recently, a fully covered metal stent (biflanged metal stent [BFMS]) has been developed for EUS‐PFD that has a high drainage effect and is anchored at both ends to prevent migration and dislocation, and it is being used in clinical practice. Once the stent is placed, the endoscope can be easily inserted and removed from the WON, allowing EN to be performed efficiently. Necrosectomy can be performed with good vision and fewer procedures, possibly contributing to reduced adverse events. The first developed BFMS was the AXIOS stent (Xlumena Corporation, now Boston Scientific Corporation), developed by Binmoeller et al. in 2011 [40], and the first clinical report was published by Itoi et al. in 2012 [41]. There are currently several BFMSs with different anchor shapes and delivery systems. They can be categorized into two main types: flared metal stents (Nagi stent; Taewoong) [42, 43, 44] and lumen‐apposing metal stents (LAMSs) (AXIOS stent; Boston Scientific and SPAXUS stent; Taewoong) [45]. In recent years, a BFMS combining the advantages of both types has also been developed, and its usefulness is expected (Plumber stent; MI Tech) [46].

Additionally, an integrated one‐step stent delivery system (HOT‐AXIOS System; Boston Scientific) capable of performing electric puncture, tract dilation, and stent deployment simultaneously has been developed. These devices enable stent placement at the bedside without fluoroscopy [47, 48, 49, 50]. In a review involving many cases, the stent placement success rate was reported to be 98% to 100%, with good clinical response rates (pancreatic pseudocyst [PPC] 84%–100%, WON 78%–90%) [51]. Since LAMS has a short stent length and requires space for deployment, it is indicated for symptomatic PPC ≥6 cm or symptomatic WON with ≥70% liquid content adherent to the gastric or intestinal wall. However, the advantages of LAMS are most evident in WON with low fluid and high necrotic content, requiring additional necrosectomy [52, 53, 54]. Techniques for safe stent placement in such cases have also been reported [55].

Several studies have reported clinical outcomes comparing LAMS and DPS use for endoscopic treatment of pancreatic fluid collections, including local complications after pancreatitis [56, 57, 58, 59, 60, 61, 62, 63, 64, 65, 66]. In the Japanese Clinical Guidelines for Acute Pancreatitis, a meta‐analysis of 10 retrospective cohort studies showed that, despite a larger number of difficult WON cases in the LAMS group, the clinical success rate was higher. Thus, LAMS is proposed for difficult cases requiring additional treatment [3]. In recent years, several RCTs comparing clinical outcomes of LAMS and DPS for WON have also been reported [64, 67, 68, 69] [Table 1]. Although lesion difficulty varied somewhat between studies, the procedure time was shorter with LAMS, but clinical success, additional EN, and adverse event rates were comparable. One disadvantage of LAMS is the high cost. However, a Japanese cost analysis found no significant difference in total treatment cost, and LAMS may be more cost‐effective due to efficiency in difficult cases requiring additional procedures like necrosectomy [56]. Because treatment is efficient, the overall adverse event rate tends to be lower in the LAMS group, but LAMS‐specific events remain a concern. Bleeding from the cyst wall and pseudoaneurysm rupture may occur more frequently, requiring caution [70, 71]. In the first RCT (30 cases each), clinical success rates were equivalent, but the LAMS group had more bleeding complications [64]. Rapid cavity shrinkage from effective drainage and mechanical stimulation by the stent tip against the opposite cyst wall may be the cause. Furthermore, long‐term LAMS placement may result in embedding into the GI mucosa, making removal difficult—a condition called buried stent syndrome [72, 73, 74]. While further large‐scale studies including RCTs are needed, early LAMS removal (within 2–4 weeks) is recommended to reduce adverse events. It is important to understand the pros and cons of LAMS and DPS for EUS‐PFD and select appropriately based on the case.

**TABLE 1:** Randomized controlled trials comparing the double pigtail plastic stent and lumen‐apposing metal stent for endoscopic ultrasound (EUS)‐guided pancreatic fluid collection drainage.

Author	Year	Number of cases (LAMS vs. D‐PS)	Size of PFC (mm)	Mean procedure time (min)	Additional EN rate (%)	Mean number of EN sessions	Clinical success rate (%)	Procedure‐related adverse event rate (%)	Mortality rate (%)	Bleeding‐related adverse event rate (%)
Bang	2019	31 vs. 29	102 vs 107	18 vs. 41.6	12.9 vs. 20.7	0.13 vs. 0.28	93.5 vs. 96.6	41.9 vs. 20.7	3.2 vs. 3.4	16.1 vs. 3.4
Boxhorn	2023	53 vs. 51	N/A	N/A	64.2 vs. 52.9	2.4 vs. 1.8	88.7 vs. 82.4	24.5 vs. 21.6	11.3 vs. 17.6	17.0 vs. 21.6
Karstensen	2023	20 vs. 22	297 vs. 215	30 vs. 59	40.0 vs. 50.0	5.3 vs. 4.5	90.0 vs. 95.5	5.0 vs. 9.1	5.0 vs. 4.5	0 vs. 4.5
Gornals	2024	33 vs. 31	110 vs 118	35 vs. 45	N/A	2.0 vs. 2.0	89.2 vs. 84.6	36 vs. 45	6.0 vs. 6.0	15.0 vs. 10.0

D‐PS, double pigtail plastic stent; EN, endoscopic necrosectomy; LAMS, lumen‐apposing metal stent; N/A, not available; PFC, pancreatic fluid collection.

### 2.4 Treatment of Cases With Complicated Morphological Features

**FIGURE 2:**
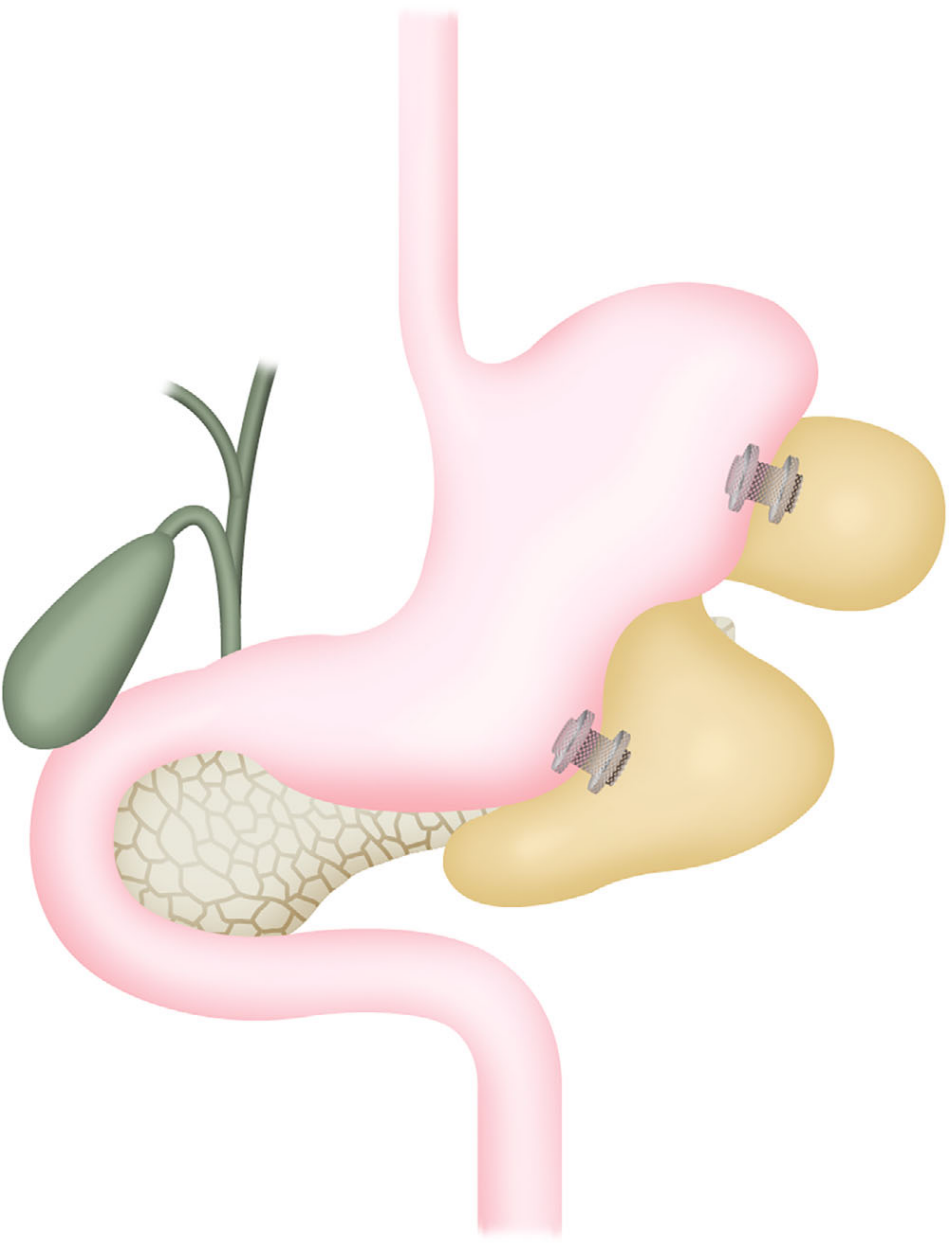
Schema showing multiple transluminal gateway drainage using a lumen‐apposing metal stent.

**FIGURE 3:**
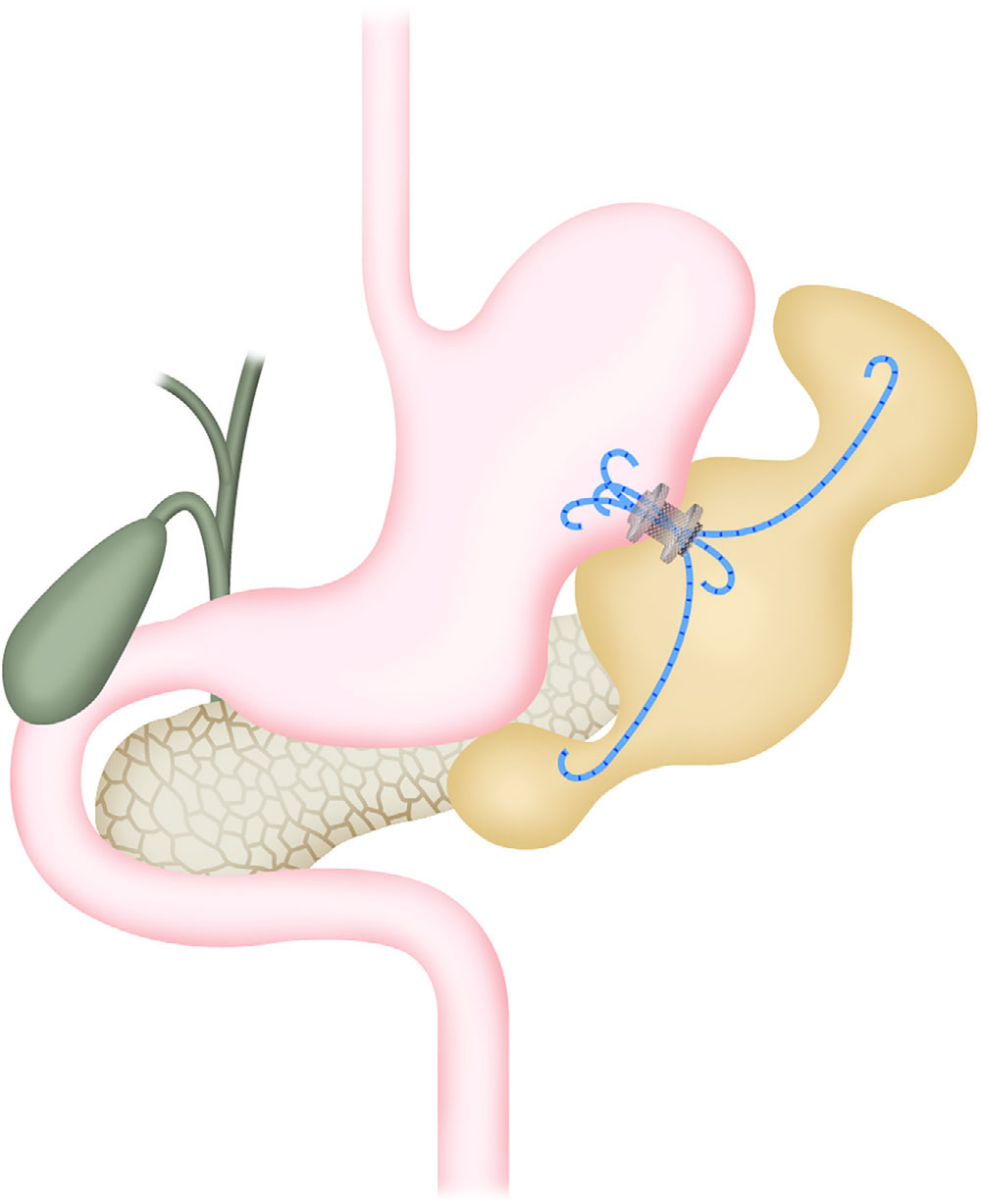
Schema showing single transluminal gateway transcystic multiple drainages after endoscopic ultrasound (EUS)‐guided drainage using a lumen‐apposing metal stent for complicated multilocular walled‐off necrosis.

WON often shows a simple unilocular form, but sometimes presents as multilocular and complicated [75]. Basically, each cavity is connected, but when the connection is narrow, even if drainage and necrosectomy are performed on the main cavity around the pancreas, the separated sub‐cavity may not drain properly, making infection control difficult. As a treatment for such multifocal WON, the usefulness of the multiple transluminal gateway technique, which involves performing EUS‐TD at multiple separate sites and additional EN from multiple tracts as necessary, has been reported [76] (Figure 2). This technique enhances the efficacy of irrigation through a nasal drainage tube and reduces the number of additional procedures, including EN, resulting in favorable outcomes. On the other hand, when sub‐cavities are located away from the gastrointestinal tract, such as in the splenic hilum or pelvic area, and direct drainage through the gastrointestinal tract is difficult, an approach called single transluminal gateway transcystic multiple drainages is useful. This involves seeking communication between the main cavity and sub‐cavities and placing a double pigtail plastic stent or a nasocystic drainage tube through this communication for drainage [77] (Figure 3). By using these techniques, improvement in the outcome of endoscopic therapy alone can be expected. In our experience, 93% of infectious WONs were successfully treated with endoscopic therapy alone [75]. However, drainage is not always necessary for all sub‐cavities of multifocal WON. If the sub‐cavities are small, they will disappear if drainage of the main lesion is adequate. In our analysis, sub‐cavities larger than 65 mm^3^ (approximately 5 cm) are likely to require drainage, and additional drainage should be actively considered [75].

### 2.5 Additional Percutaneous Approach

Although percutaneous drainage is a minimally invasive and effective treatment, it has disadvantages such as recurrence, refractory skin fistulas, and a tendency to decrease patients' ADL, and therefore is not currently the first choice approach [78]. However, it is often useful in cases of huge WON extending from the retroperitoneal cavity to the left or right pelvis. Inserting an endoscope into the pelvic cavity through a narrow tract from the peripancreatic cavity via the gastrointestinal tract is inefficient and increases the risk of adverse events. By combining early percutaneous interventions with endoscopic treatments (hybrid approach or dual modality approach), rather than sticking to endoscopic treatments alone, it is expected that the number of procedures, including necrosectomy, can be reduced and the risk of adverse events lowered [79, 80, 81].

**FIGURE 4:**
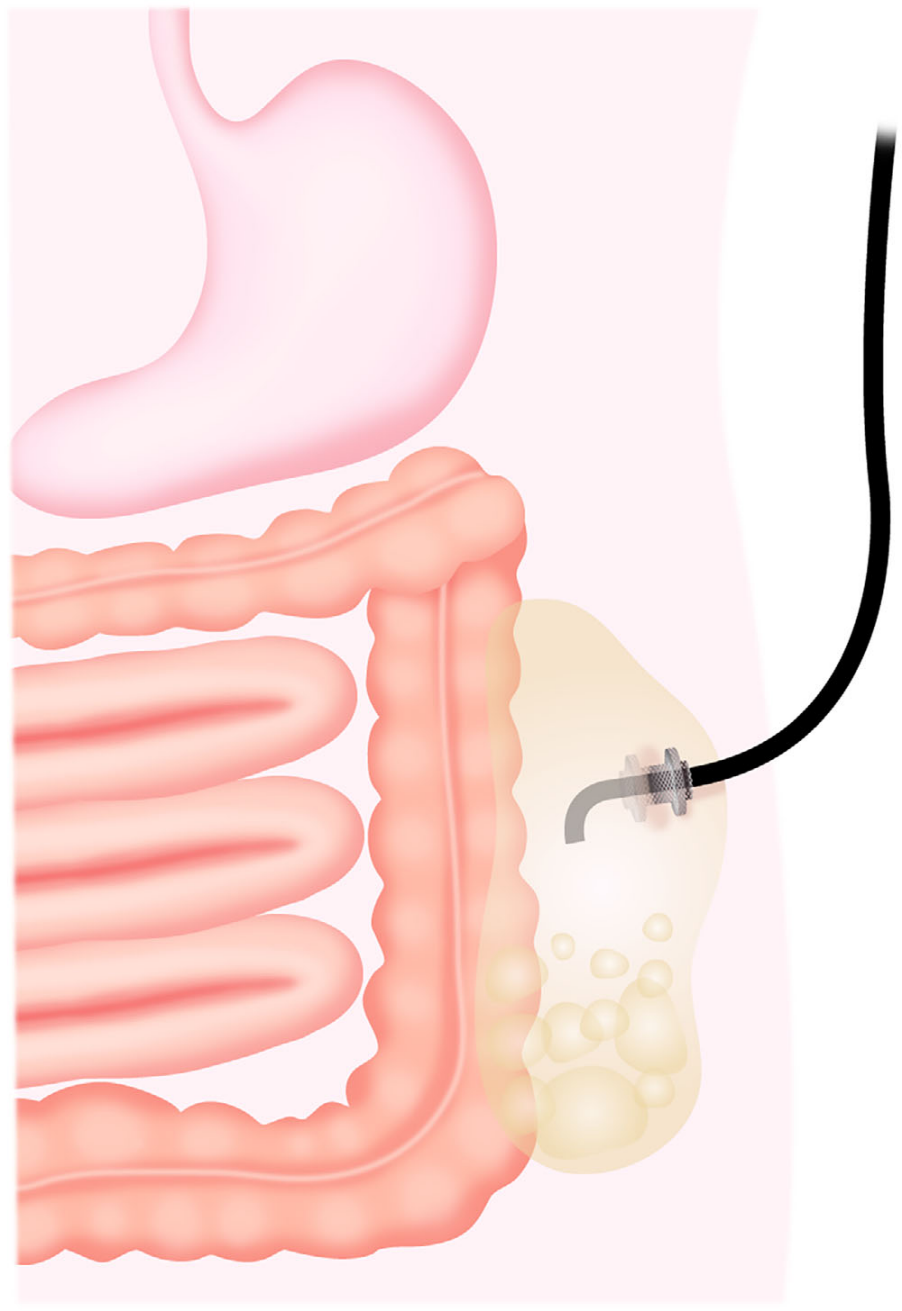
Schema showing percutaneous endoscopic necrosectomy for the pelvic cavity of walled‐off necrosis.

In 117 cases of WON with widespread lesions, it has been reported that early use of this combination therapy resulted in a favorable outcome with a clinical response rate of 88%, and that percutaneous drainage tubes were removed in all cases after treatment without the occurrence of refractory skin fistulas [82]. Furthermore, for WON cavities close to the abdominal wall, a large‐diameter metal stent is placed via a percutaneous tract, and an endoscope is inserted through the lumen of the stent to perform EN. This procedure, called percutaneous EN (PEN), has also been developed [83, 84, 85, 86] (Figure 4). After treatment, there is concern about skin fistula formation after stent removal, but if there are no nutritional concerns, granulation tissue will grow immediately, and the skin fistula will close. There have been only a few case reports, and further evaluation of safety and efficacy is necessary. However, this treatment can be performed with intravenous anesthesia alone, similar to conventional percutaneous procedures, and is a minimally invasive treatment. It has been reported that 79.2% of 53 refractory cases of WON were successfully treated with PEN [87].

### 2.6 Surgical Necrosectomy

In cases that are difficult to treat with endoscopic or percutaneous approaches, surgical necrosectomy should be considered. Surgical necrosectomy is mainly divided into open necrosectomy and laparoscopic necrosectomy using retroperitoneal approaches such as VARD. According to the results of a meta‐analysis using previous cohort studies, both the mortality rate and morbidity rate were significantly lower with the retroperitoneal approach, and the guidelines also recommend selecting retroperitoneal necrosectomy [3, 88, 89].

### 2.7 Disconnected Pancreatic Duct Syndrome

In some cases, diffuse pancreatic necrosis causes pancreatic duct rupture at the level of the main pancreatic duct, resulting in loss of connection between the head and tail of the pancreatic duct and continued leakage of pancreatic fluid into the abdominal, thoracic, or retroperitoneal cavity. This condition is referred to as disconnected pancreatic duct syndrome (DPDS) and is reported to occur in approximately 16%–23% of WON cases [90, 91]. DPDS has been associated with symptoms such as abdominal pain and nausea, prolonged hyperamylasemia, and recurrence due to spontaneous dislodgement or obstruction of drainage tubes after WON treatment. In the long term, it affects pancreatic function, such as deterioration of digestive function and carbohydrate metabolism. Bang et al. reported that evaluating the main pancreatic duct during EUS‐TD and following the main pancreatic duct in the tail toward the head, if the duct was found to enter the WON cavity and could no longer be followed, DPDS was considered to be present in nearly 100% of cases, and this finding was useful for selecting drainage strategies and considering stent removal after treatment [92]. For the diagnosis and treatment of DPDS, pancreatography by ERCP is performed to evaluate the pancreatic duct, and transpapillary treatment involving the placement of a pancreatic duct stent to bridge the ruptured head and tail is useful. However, in cases where the duct is completely disconnected, treatment is often difficult. The success rate of stent placement is high at 92% in partial disruptions, but only 20% in complete disconnections [93]. As an alternative treatment for cases difficult to manage with transpapillary treatment, EUS‐guided percutaneous endoscopic drainage and surgical treatments such as distal pancreatectomy and pancreaticojejunostomy have been attempted [94, 95].

## 3 Conclusion

This review summarizes the latest interventional treatments, focusing on the endoscopic step‐up approach for WON after acute pancreatitis. The use of dedicated large‐bore metal stents (mainly LAMS) and various techniques has improved the outcomes of endoscopic treatment alone. However, it is important to consider a comprehensive treatment strategy that includes percutaneous approaches and surgical approaches, rather than focusing solely on endoscopic treatment.

## Conflicts of Interest

Takayoshi Tsuchiya has received honoraria for lectures from Boston Scientific. Takao Itoi has received consulting fees from Boston Scientific. Takao Itoi is the Editor‐in‐Chief of DEN Open. Shuntaro Mukai is an Associate Editor of DEN Open.

## Ethics Statement

Approval of the research protocol by an Institutional Review Board: N/A.

## Consent

Written informed consent for the procedure was obtained from all patients.

## Clinical Trial Registration

N/A
